# Association between indicators of visceral lipid accumulation and infertility: a cross-sectional study based on U.S. women

**DOI:** 10.1186/s12944-024-02178-x

**Published:** 2024-06-13

**Authors:** Chenyuan Deng, Xinpeng Ke, Liangcai Lin, Yong Fan, Chaohui Li

**Affiliations:** Department of Obstetrics and Gynecology, Guangdong Provincial Key Laboratory of Major Obstetric Diseases, Guangdong Provincial Clinical Research Center for Obstetrics and Gynecology, Guangdong-Hong Kong-Macao Greater Bay Area Higher Education Joint Laboratory of Maternal-Fetal Medicine, The Third Affiliated Hospital of Guangzhou Medical University, Guangzhou, Guangdong China

**Keywords:** Lipid accumulation, Obesity, Infertility, Reproduction, NHANES

## Abstract

**Background:**

Evidence on the association between visceral lipid accumulation and infertility remains limited and controversial. Therefore, the current investigation is the first investigation to unveil this correlation by utilizing novel indicators of visceral lipid accumulation.

**Methods:**

The present study utilized the NHANES 2013–2020 dataset. Researchers utilized multiple logistic regression, smoothed curve fitting, and subgroup analysis to investigate the associations of waist circumference (WC), metabolic score for visceral fat (METS-VF), lipid accumulation product (LAP), visceral adiposity index (VAI) with infertility. Additionally, the eXtreme Gradient Boosting (XGBoost) algorithm model was utilized to evaluate the relative importance of the factors.

**Results:**

After adjusting for potential factors that could influence the results, researchers discovered that all these four indicators of visceral lipid accumulation exhibited strong positive correlations with the probability of infertility. The subgroup analysis demonstrated that the correlations remained consistent in the majority of subgroups (*P* for interaction > 0.05). The results of XGBoost algorithm model indicate that METS-VF is the most meaningful factor in infertility. The ROC curve research revealed that while METS-VF had the greatest AUC values, there was no variation in the AUC value of different markers of visceral fat accumulation (*P* > 0.05).

**Conclusions:**

The present investigation discovered that increased WC, METS-VF, LAP, and VAI were associated with a heightened prevalence of infertility.

**Supplementary Information:**

The online version contains supplementary material available at 10.1186/s12944-024-02178-x.

## Introduction

Infertility is a medical condition that prevents pregnancy after 12 months of consistent, unprotected sexual intercourse [1]. Infertility, a prevalent issue in reproductive health on a global scale, impacts a substantial number of individuals, estimated at 186 million worldwide [2]. In the United States, a noteworthy proportion of women within the childbearing age bracket, roughly 12.7%, actively pursue infertility therapy annually [3]. Secondary infertility caused by reproductive system infections is the most common kind of female infertility [1]. Zhang et al. conducted a study that further validated pelvic inflammatory illness as the primary factor contributing to secondary infertility among women in local population [4]. A separate study including 71 American women has corroborated that genitourinary Chlamydia trachomatis is a significant factor contributing to female infertility [5]. Moreover, many medical disorders, including endometriosis and thyroid dysfunction, have been identified as potential factors contributing to infertility [6, 7]. There is evidence suggesting that lifestyle and environmental factors also exert an influence on the reproductive health of women via neuroendocrine pathways [8]. These identified risk factors potentially facilitate the production of an excessive amount of free radicals within the organism, hence initiating an oxidative state that poses a threat to the reproductive health of the organism [9]. Currently, making changes to one’s lifestyle is the most fundamental and economical approach to treating infertility. The Dietary Inflammatory Index (DII) measures the degree of dietary inflammation within the body. Vahid et al. surveyed 135 Iranian women and discovered that women with elevated DII scores have an increased likelihood of miscarriage in comparison with women adhering to an anti-inflammatory diet [10]. An empirical study with a sample of 577 women demonstrated that enhancing fertility can be achieved by diminishing the consumption of treats and drinks with a high-calorie content, alongside augmenting exercise [11]. Other primary methods used to treat infertility also include ovarian stimulation, in vitro fertilization, psychotherapy, and stem cell therapy [3, 12, 13]. However, given the intricate nature and exorbitant expenses associated with treatments of infertility, treating infertility remains to be a significant challenge in the realm of human reproductive health. Therefore, it is imperative to carry out additional research on the risk factors of infertility to offer novel insights and approaches for the prevention and management of infertility.

Obesity has evolved into an epidemic worldwide health disorder, impacting a huge number of individuals globally. According to dependable data, the prevailing prevalence of overweight or obesity is approximated to affect in excess of 1.1 billion individuals and approximately 10% of children worldwide [14]. Additionally, it should be noted that obesity approximately contributes to a range of 0.7–2.8% of a nation’s overall healthcare costs [15]. Numerous research has provided substantial evidence indicating a positive correlation between obesity and the heightened susceptibility to cardiovascular disorders, asthma, and type 2 diabetes [16–18]. During the past couple of decades, the influence of being overweight on female fertility has also received much scrutiny. A Danish investigation revealed that women with extreme obesity are far more likely to experience adverse pregnancy outcomes, such as gestational diabetes and pre-eclampsia. Furthermore, their infants exhibited significantly higher rates of obesity [19]. In addition, Fedorcsák et al. substantiated that women who are obese possess a reduced number of oocytes and are more susceptible to a miscarriage in a study including 383 women [20]. Consequently, more and more women acknowledge that maintaining a healthy weight can successfully promote women’s reproductive health. A prospective study conducted by Clark et al. demonstrated that overweight women with infertility who engaged in physical exercise to lose weight saw a restoration of ovulation and a decrease in miscarriage rates [21]. Another study conducted by Christinajoice et al. including 45 female participants demonstrated that bariatric surgery significantly improved the reproductive health of subjects [22]. Obesity raises the likelihood of infertility in women and has negative effects on assisted reproductive technologies [23, 24]. Nevertheless, most previous research on the correlation between obesity and infertility has predominantly relied on body mass index (BMI) as the primary indicator of obesity. It’s controversial because research has demonstrated that individuals with a high amount of visceral fat are at a greater risk of experiencing heart-related problems, regardless of whether their BMI is considered normal or excessive [25]. Therefore, relying solely on BMI for evaluating obesity is not reasonable, and taking into account central obesity offers a broader comprehension of the health concerns associated with obesity. While waist circumference (WC) is widely employed to measure central adiposity, it is important to consider that the accuracy of WC may be influenced by the subject’s height and BMI [26, 27]. Therefore, to address the limitations of WC, the researchers suggest developing novel indicators to accurately measure visceral lipid accumulation. The metabolic score for visceral fat (METS-VF), lipid accumulation product (LAP), and visceral adipose index (VAI) are reliable indicators used to measure the accumulation of visceral lipids. Numerous prior research has established a robust correlation between these indicators and various disorders [28–30]. Considering the easy accessibility of data for calculating these indicators, it is anticipated that they will assume a significant role in the assessment of a greater number of diseases in the future. No research has investigated the significance of indicators of visceral lipid accumulation in relation to female infertility. This study is the inaugural investigation to reveal the associations between these indicators and infertility. By doing so, it could offer a more effective approach for the future management and therapy for infertility in both affected individuals and those who may be susceptible. Additionally, it will provide novel perspectives on the impact of lipids on infertility. The researchers hypothesized that all four indicators of visceral lipid accumulation examined in this study would exhibit substantial correlations with infertility.

## Methods

### Data sources for the study

The National Health and Nutrition Examination Survey (NHANES) 2013–2020 is the resource of the data for the current analysis. The National Center for Health Statistics (NCHS) Ethics Review Board granted authorization for the human subjects in NHANES, and every single participant provided their informed permission. Demographic data, laboratory data, examination data, and questionnaire data were all gathered as part of this survey. These data were then further analyzed and used to research risk factors of various disorders. This study employed a sophisticated multi-stage probabilistic methodology, which ensured that the sample used was representativity and accurate.

### Study population

**Fig. 1:**
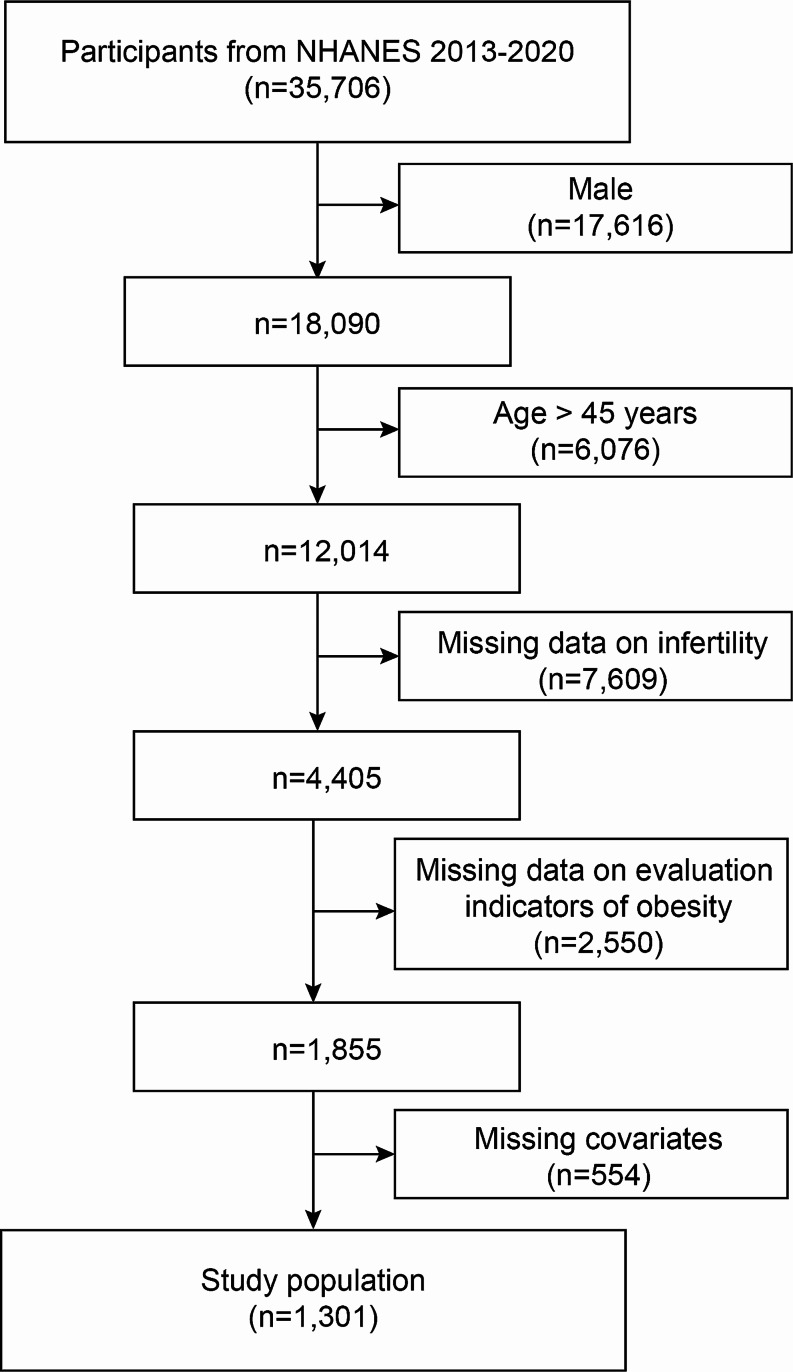
Flowchart for selecting samples from NHANES 2013–2020. NAHNES, National Health and Nutrition Examination Survey

35,706 participants from the NHANES 2013–2020 were initially added. To choose the ones who best matched the current study, researchers further evaluated these people. First of all, subjects who were 45 years of age or older (*n* = 6,076) was ineligible as well as male subjects (*n* = 17,616). Participants who lacked indicators of visceral lipid accumulation data (*n* = 2,550), infertility data (*n* = 7,609), and covariate data (*n* = 554) were also eliminated. In the end, 1,301 qualified female individuals took part in the study (Fig. 1).

### Definition of exposure variables

Researchers considered METS-VF, WC, VAI, and LAP as exposure variables. Their calculation is determined by the following formula [31]. At the Mobile Examination Center (MEC), professional health technicians measure individuals’ BMI, WC, and height carefully. High-density lipoprotein cholesterol (HDL-C) and triglycerides (TG) data were obtained using the Cobas 6000 Chemistry Analyzer. The Roche/Hitachi Cobas C Chemistry Analyzer - C311 yielded data on fasting blood glucose (FBG).

$$\eqalign{ VAI = & {{WC\left( {cm} \right)} \over {36.58 + 1.89 \times BMI}} \times {{TG(mmol/L)} \over {0.81}} \cr & \times {{1.52} \over {HDL - C(mmol/L)}}\,{\rm{for}}\,{\rm{females;}} \cr}$$

$$LAP = (WC\left( {cm} \right) - 58) \times TG(mmol/L)\,{\rm{for}}\,{\rm{females;}}$$

$$WHtR=\frac{WC}{Height}$$

$$METS-IR=\frac{\text{l}\text{n}(2\times FBG+TG)\times BMI}{\text{l}\text{n}(HDL-C)}$$

$$\eqalign{ METS - VF & = 4.466 + 0.01 \times {\left( {{\rm{ln}}\left( {{\rm{METS}} - {\rm{IR}}} \right)} \right)^3} \cr & + 3.329 \times {\left( {{\rm{ln}}\left( {{\rm{WHtR}}} \right)} \right)^3} + 0.319 \cr & \times gender + 0.594 \times {\rm{ln}}(age) \cr}$$

Gender was presumed to be 0 because there were no male participants in this study.

### Definition of infertility

The ending variable was infertility. Through a query in the Computer Assisted Personal Interview (CAPI) system at the Mobile Examination Center (MEC), information about infertility was collected. When asked, “Have you been trying to get pregnant for one year? (RHQ074)”, individuals were categorized as having infertility if they responded positively; otherwise, they were categorized as not having infertility.

### Covariates

Researchers evaluated these variables as covariates and accounted for them during data analysis since they could potentially affect how indicators of visceral lipid accumulation and infertility are related. Covariates of the investigation included race, educational attainment, matrimonial status, family income to poverty ratio, total cholesterol, smoking, alcohol usage, physical activity, menstruation status, pelvic inflammatory disease therapy, and use of hormonal medicines. Smokers are defined as having smoked at least 100 cigarettes. Drinkers are defined as having had 4 to 5 drinks or more per day in the past 12 months. Subjects were defined as having physical exercise if they had exercised at a moderate or vigorous intensity during a typical week. You are able to locate comprehensive measurement methods for each of these variables at the NHANES Official Website.

### Methods and tools used for statistical analysis of data

Researchers used R version 3.4.3 and Empower software to assist with the present statistical investigation. Given the non-normal distribution of the continuous variables in the study, they were reported as the median (quartile 1-quartile 3). Categorical variables were represented using percentages. Additionally, the chi-square test was employed for categorical variables, while the Mann-Whitney U-test was utilized for continuous data. Three distinct models were established in the multiple logistic regression analysis to assess the correlation between the independent variables and the dependent variable. The first model did not adjust for any variable. The second model adjusted for age and race. And the third model adjusted for all covariates considered in the study, including age, race, education, marital status, family income to poverty ratio, total cholesterol, smoking, alcohol use, physical activity, menstrual status, treatment of pelvic inflammatory disease, and use of hormonal medications. Furthermore, smoothed curve fitting and threshold effect analysis were employed to explore potential non-linear relationships of exposure variables with outcome variables. In addition, researchers analyzed subgroups according to marital status, alcohol use, smoking, physical exercise, and regularity of menstruation. The researchers employed a novel approach by utilizing Python software to implement the eXtreme Gradient Boosting (XGBoost) algorithm, known for its effectiveness in machine learning models. This method was utilized to ascertain the relative significance of the chosen variables. Grid search and cross-validation techniques were utilized to optimize the XGBoost algorithm model and enhance its performance. In the XGBoost model, the adjusted hyperparameters included n_estimators, max_depth, and learning_rate. Specifically, the values for these hyperparameters were: n_estimators: 100, max_depth: 3, and learning_rate: 0.1. Researchers compared the association of different indicators of visceral lipid accumulation with visceral obesity and infertility by means of subject work characteristics (ROC) curve applying logistic regression and calculating the area under the curve (AUC). The Delong test, implemented in Medcalc statistical software, was used to compare the differences in AUC values. In the investigation, a significance level of *P* < 0.05 was used to determine statistically significant differences.

## Results

### Details of essential characteristics of participants

Table 1 provides a concise summary of the essential characteristics of the individuals involved in the study. The current study was conducted with 1,301 eligible female participants, whose median age was 33 years. Among the participants, 1,137 individuals were categorized as not having infertility, while 164 individuals met the criteria for infertility. Infertile patients exhibited distinct characteristics compared to those without infertility. They had higher median age, a greater proportion of individuals who used alcohol, and higher median values for BMI, WC, LAP, VAI, and METS-VF.

**Table 1:** Baseline population characteristics of this study population

	Total (*n* = 1,301)	No infertility (*n* = 1,137)	Infertility (*n* = 164)	*P*-value
**Age (years)**	33.00 (26.00–39.00)	32.00 (26.00–39.00)	36.00 (29.00–41.00)	< 0.001
**Family income to poverty ratio**	1.99 (0.98–3.81)	1.99 (0.97–3.79)	2.11 (1.11–3.94)	0.435
**Total cholesterol (mg/dL)** **BMI (kg/m^2)**	173.00 (153.00-199.00) 28.50 (23.30–35.10)	173.00 (153.00-199.00) 28.00 (23.00-34.40)	176.00 (152.75-198.25) 32.15 (24.60-37.88)	0.981 < 0.001
**Race**				0.506
Mexican American	188 (14.45%)	166 (14.60%)	22 (13.41%)	
Other Hispanic	124 (9.53%)	112 (9.85%)	12 (7.32%)	
Non-Hispanic White	476 (36.59%)	407 (35.80%)	69 (42.07%)	
Non-Hispanic Black	299 (22.98%)	261 (22.96%)	38 (23.17%)	
Other Race	214 (16.45%)	191 (16.80%)	23 (14.02%)	
**Education level**				0.952
Less than high school	165 (12.68%)	143 (12.58%)	22 (13.41%)	
High school	235 (18.06%)	206 (18.12%)	29 (17.68%)	
More than high school	901 (69.25%)	788 (69.31%)	113 (68.90%)	
**Marital status**				< 0.001
Married/Living with partners	730 (56.11%)	607 (53.39%)	123 (75.00%)	
Widowed/Divorced/Separated	354 (27.21%)	330 (29.02%)	24 (14.63%)	
Never married	217 (16.68%)	200 (17.59%)	17 (10.37%)	
**Alcohol use**				0.020
Yes	106 (8.15%)	85 (7.48%)	21 (12.80%)	
No	1195 (91.85%)	1052 (92.52%)	143 (87.20%)	
**Smoking**				0.227
Yes	453 (34.82%)	389 (34.21%)	64 (39.02%)	
No	848 (65.18%)	748 (65.79%)	100 (60.98%)	
**Physical activity**				0.928
Yes	631 (48.50%)	552 (48.55%)	79 (48.17%)	
No	670 (51.50%)	585 (51.45%)	85 (51.83%)	
**Regular menstruation**				0.562
Yes	1167 (89.70%)	1022 (89.89%)	145 (88.41%)	
No	134 (10.30%)	115 (10.11%)	19 (11.59%)	
**Treated for pelvic inflammatory disease**				0.663
Yes	70 (5.38%)	60 (5.28%)	10 (6.10%)	
No	1231 (94.62%)	1077 (94.72%)	154 (93.90%)	
**Used hormonal drugs**				0.358
Yes	54 (4.15%)	45 (3.96%)	9 (5.49%)	
No	1247 (95.85%)	1092 (96.04%)	155 (94.51%)	
**Indicators of visceral lipid accumulation**				
**WC**	94.20 (81.50-108.60)	93.40 (80.70-107.20)	101.10 (86.22-117.33)	< 0.001
**LAP**	30.34 (14.53–54.69)	29.14 (13.52–53.12)	37.41 (22.30-67.91)	< 0.001
**VAI**	1.12 (0.68–1.81)	1.09 (0.66–1.79)	1.30 (0.87–1.90)	0.002
**METS-VF**	6.49 (5.83–6.95)	6.44 (5.78–6.93)	6.76 (6.22–7.05)	< 0.001

BMI, body mass index; WC, waist circumference; LAP, lipid accumulation product; VAI, visceral adiposity index; METS-VF, metabolism score for visceral fat

### Correlations between indicators of visceral lipid accumulation and infertility

In the findings devoid of confounding adjustments, a positive correlation was discerned between WC, METS-VF, LAP, VAI, and the prevalence of infertility (Table 2). After adjusting for all other confounders, the prevalence of infertility demonstrated an escalation of 2% aligned with each unit rise in WC (Odds Ratio [OR] = 1.02, 95% Confidence Interval [CI]: 1.01–1.03). Drawing a parallel, the prevalence of infertility augmented by 82% (OR = 1.82, 95% CI: 1.39–2.40) concomitant with each unit climb in METS-VF, by 1% (OR = 1.01, 95% CI: 1.00-1.01) recurrent with each unit ascension in LAP, and by 12% (OR = 1.12, 95% CI: 1.03–1.23) with each unit increase in VAI. Furthermore, the calculating formula involved converting the centimeter (CM) units to decimeters (DM) in order to amplify the effect size values of WC and LAP with infertility. Once again, multiple logistic regression analysis was employed to examine the relationship of WC and LAP with infertility. The prevalence of infertility increased by 18% for each whole-number rise in WC (OR = 1.18, 95% CI: 1.09–1.29) and by 6% for each whole-number increase in LAP (OR = 1.06, 95% CI: 1.03–1.10) (Supplementary 1). The association between these visceral obesity proxies and infertility was further explored after converting WC, METS-VF, LAP, and VAI from continuous to categorical variables (tertiles). Within the model that has been fully adjusted, the prevalence of experiencing infertility was 202% higher in those with the highest tertiles of WC in comparison to subjects with the lowest tertiles of WC (OR = 2.02, 95% CI:1.28–3.18). The prevalence of infertility in the highest tertiles of METS-VF was 248% of that in the lowest tertiles of METS-VF (OR = 2.34, 95% CI:1.46–3.77). The prevalence of infertility in the highest tertiles of LAP was 234% of the risk in the lowest tertiles of LAP (OR = 1.82, 95% CI:1.39–2.40). The prevalence of infertility in the highest tertiles of VAI was 210% of the lowest tertiles of VAI (OR = 2.10, 95% CI:1.31–3.37). All *P*-trends were statistically significant.

**Table 2:** Exploring the associations between indicators of visceral lipid accumulation and infertility using multivariate logistic regression

Exposure	OR (95% CI), *P*-value		
	Model 1	Model 2	Model 3
WC	1.02 (1.01, 1.02) < 0.0001	1.01 (1.01, 1.02) 0.0004	1.02 (1.01, 1.03) 0.0001
WC tertiles			
T1	1.00 (Reference)	1.00 (Reference)	1.00 (Reference)
T2	1.25 (0.80, 1.94) 0.3201	1.16 (0.73, 1.82) 0.5286	1.17 (0.73, 1.86) 0.5154
T3	2.05 (1.36, 3.09) 0.0006	1.86 (1.21, 2.86) 0.0047	2.02 (1.28, 3.18) 0.0025
*P*-trend	0.0003	0.0023	0.0011
METS-VF	1.75 (1.37, 2.25) < 0.0001	1.76 (1.37, 2.27) < 0.0001	1.82 (1.39, 2.40) < 0.0001
METS-VF tertiles			
T1	1.00 (Reference)	1.00 (Reference)	1.00 (Reference)
T2	1.69 (1.08, 2.64) 0.0204	1.76 (1.12, 2.77) 0.0140	1.71 (1.07, 2.73) 0.0257
T3	2.31 (1.50, 3.53) 0.0001	2.35 (1.52, 3.64) 0.0001	2.48 (1.55, 3.96) 0.0001
*P*-trend	0.0001	0.0001	0.0001
LAP	1.00 (1.00, 1.01) 0.0010	1.00 (1.00, 1.01) 0.0070	1.01 (1.00, 1.01) 0.0005
LAP tertiles			
T1	1.00 (Reference)	1.00 (Reference)	1.00 (Reference)
T2	1.64 (1.06, 2.55) 0.0277	1.53 (0.98, 2.40) 0.0643	1.68 (1.06, 2.67) 0.0277
T3	2.20 (1.44, 3.36) 0.0003	1.97 (1.27, 3.05) 0.0024	2.34 (1.46, 3.77) 0.0004
*P*-trend	0.0004	0.0037	0.0008
VAI	1.12 (1.03, 1.21) 0.0059	1.10 (1.02, 1.20) 0.0170	1.12 (1.03, 1.23) 0.0086
VAI tertiles			
T1	1.00 (Reference)	1.00 (Reference)	1.00 (Reference)
T2	2.12 (1.37, 3.28) 0.0008	2.07 (1.33, 3.21) 0.0012	2.21 (1.41, 3.48) 0.0006
T3	2.03 (1.31, 3.16) 0.0015	1.89 (1.20, 2.96) 0.0056	2.10 (1.31, 3.37) 0.0022
*P*-trend	0.0106	0.0331	0.0149

Model 1, crude model;

Model 2, adjusted for age, race;

Model 3, adjusted for age, race, education, marital status, family income to poverty ratio, total cholesterol, smoking, alcohol use, physical activity, menstrual status, treatment of pelvic inflammatory disease, and use of hormonal medications

Age was not adjusted in the evaluation of the correlation between METS-VF and infertility

OR, Odds ratio; CI, Confidence Interval; WC, waist circumference; METS-VF, metabolism score for visceral fat; LAP, lipid accumulation product; VAI, visceral adiposity index

**Fig. 2:**
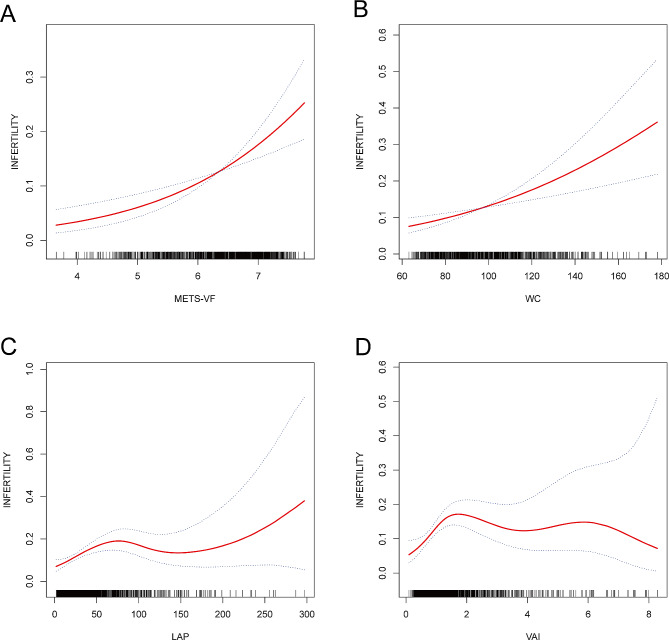
Association between different evaluation indicators of central obesity and infertility. (**A**) Association between METS-VF and infertility; (**B**) Association between WC and infertility; (**C**) Association between LAP and infertility; (**D**) Association between VAI and infertility. The solid red line represents the smooth curve fit between variables. Blue bands represent the 95% of confidence interval from the fit. WC, waist circumference; METS-VF, metabolism score for visceral fat; LAP, lipid accumulation product; VAI, visceral adiposity index

Figure 2 offers a visual representation of the results derived from applying a procedure to fit a smoothed curve, while the outcomes of the threshold effect analysis are displayed in Table 3. After adjusting for all covariates, researchers noticed linear correlations of WC and METS-VF with infertility. However, the correlations of LAP and VAI with infertility were not linear. The additional computations revealed that the breakpoints of the correlation between LAP and infertility and the correlation between VAI and infertility were determined to be 28.8 and 1.25, respectively. Statistically significant associations of LAP and VAI with infertility were observed on the left side of the breakpoints. Whereas, on the right side of the breakpoint, researchers observed no statistically significant correlations.

**Table 3:** Threshold effects of evaluation indicators of central obesity on infertility using a two-stage linear regression model

	WC	METS-VF	LAP	VAI
**Fitting by standard linear model**				
OR (95% CI), *P*-value	1.02 (1.01, 1.03) 0.0001	1.82 (1.39, 2.40) < 0.0001	1.00 (1.00, 1.01) 0.0184	1.12 (1.03, 1.23) 0.0086
**Fitting by two-piecewise linear model**				
Inflection point (K)	118.5	7	28.8	1.25
OR1 (95% CI), *P*-value (< K)	1.02 (1.01, 1.04) 0.0007	1.98 (1.41, 2.78) < 0.0001	1.05 (1.02, 1.07) 0.0011	3.73 (1.94, 7.20) < 0.0001
OR2 (95% CI), *P*-value (≥ K)	1.00 (0.98, 1.03) 0.8314	1.00 (0.23, 4.33) 0.9961	1.00 (1.00, 1.01) 0.6307	0.89 (0.74, 1.07) 0.2158
Log likelihood ratio	0.219	0.405	0.002	< 0.001

All covariates had been adjusted

OR, Odds ratio; CI, Confidence Interval; WC, waist circumference; METS-VF, metabolism score for visceral fat; LAP, lipid accumulation product; VAI, visceral adiposity index

### Details of subgroup analysis

With the aim of determining the stability of the correlations of WC, METS-VF, LAP, and VAI with infertility in the population, additional subgroup analyses were conducted. Subgroup analyses were stratified by marital status, smoking habits, level of physical activity, and regular menstruation. The outcomes of these subgroup analyses, adjusting for all confounders, are displayed in Table 4. The results of the current investigation suggested that the correlations of WC, METS-VF, LAP, and VAI with infertility remained consistent throughout various populations (*P* for interaction > 0.05).

**Table 4:** Results of subgroup analysis

Subgroup	WC, OR (95% CI)	*P* for interaction
**Marital status**		0.1084
Married/Living with partners	1.02 (1.01, 1.04), < 0.0001	
Widowed/Divorced/Separated	1.02 (1.00, 1.04), 0.1294	
Never married	0.99 (0.96, 1.02), 0.5425	
**Alcohol use**		0.3780
Yes	1.00 (0.97, 1.04), 0.7859	
No	1.02 (1.01, 1.03), < 0.0001	
**Smoking**		0.2775
Yes	1.01 (1.00, 1.02), 0.1011	
No	1.02 (1.01, 1.03), 0.0002	
**Physical activity**		0.9982
Yes	1.02 (1.00, 1.03), 0.0080	
No	1.02 (1.01, 1.03), 0.0040	
**Regular menstruation**		0.6133
Yes	1.02 (1.01, 1.03), < 0.0001	
No	1.01 (0.98, 1.04), 0.4460	
**Subgroup**	**METS-VF, OR (95% CI)**	***P *** **for interaction**
**Marital status**		0.1188
Married/Living with partners	2.27 (1.59, 3.23), < 0.0001	
Widowed/Divorced/Separated	1.50 (0.79, 2.85), 0.2181	
Never married	0.99 (0.47, 2.09), 0.9752	
**Alcohol use**		0.2141
Yes	1.22 (0.61, 2.45), 0.5752	
No	2.00 (1.48, 2.70), < 0.0001	
**Smoking**		0.0558
Yes	1.29 (0.86, 1.94) 0.2103	
No	2.17 (1.49, 3.16) < 0.0001	
**Physical activity**		0.9287
Yes	1.80 (1.22, 2.65), 0.0028	
No	1.84 (1.27, 2.67), 0.0012	
**Regular menstruation**		0.2353
Yes	1.97 (1.47, 2.65), < 0.0001	
No	1.20 (0.56, 2.55), 0.6409	
**Subgroup**	**LAP, OR (95% CI)**	***P *** **for interaction**
**Marital status**		0.8940
Married/Living with partners	1.01 (1.00, 1.01), 0.0018	
Widowed/Divorced/Separated	1.01 (1.00, 1.01), 0.0550	
Never married	1.00 (0.99, 1.02), 0.6027	
**Alcohol use**		0.8507
Yes	1.01 (1.00, 1.01), 0.2723	
No	1.01 (1.00, 1.01), 0.0008	
**Smoking**		0.7334
Yes	1.01 (1.00, 1.01), 0.0080	
No	1.01 (1.00, 1.01), 0.0132	
**Physical activity**		
Yes	1.00 (1.00, 1.01), 0.1014	0.7597
No	1.01 (1.00, 1.01), 0.0023	
**Regular menstruation**		0.6000
Yes	1.01 (1.00, 1.01), 0.0006	
No	1.01 (1.00, 1.02), 0.1148	
**Subgroup**	**VAI, OR (95% CI)**	***P *** **for interaction**
**Marital status**		0.4960
Married/Living with partners	1.11 (1.00, 1.23), 0.0541	
Widowed/Divorced/Separated	1.20 (0.98, 1.47), 0.0760	
Never married	1.46 (0.91, 2.34), 0.1169	
**Alcohol use**		0.6935
Yes	1.11 (0.94, 1.32), 0.2027	
No	1.16 (1.05, 1.28), 0.0050	
**Smoking**		0.5514
Yes	1.09 (0.98, 1.22), 0.0962	
No	1.16 (1.00, 1.34), 0.0548	
**Physical activity**		0.3526
Yes	1.04 (0.89, 1.22), 0.5907	
No	1.14 (1.02, 1.28), 0.0180	
**Regular menstruation**		0.1083
Yes	1.12 (1.03, 1.23), 0.0125	
No	1.59 (1.06, 2.37), 0.0240	

All covariates had been adjusted

OR, Odds ratio; CI, Confidence Interval; WC, waist circumference; METS-VF, metabolism score for visceral fat; LAP, lipid accumulation product; VAI, visceral adiposity index

### Assessing the relative importance of variables in the study through XGBoost algorithm modeling

**Fig. 3:**
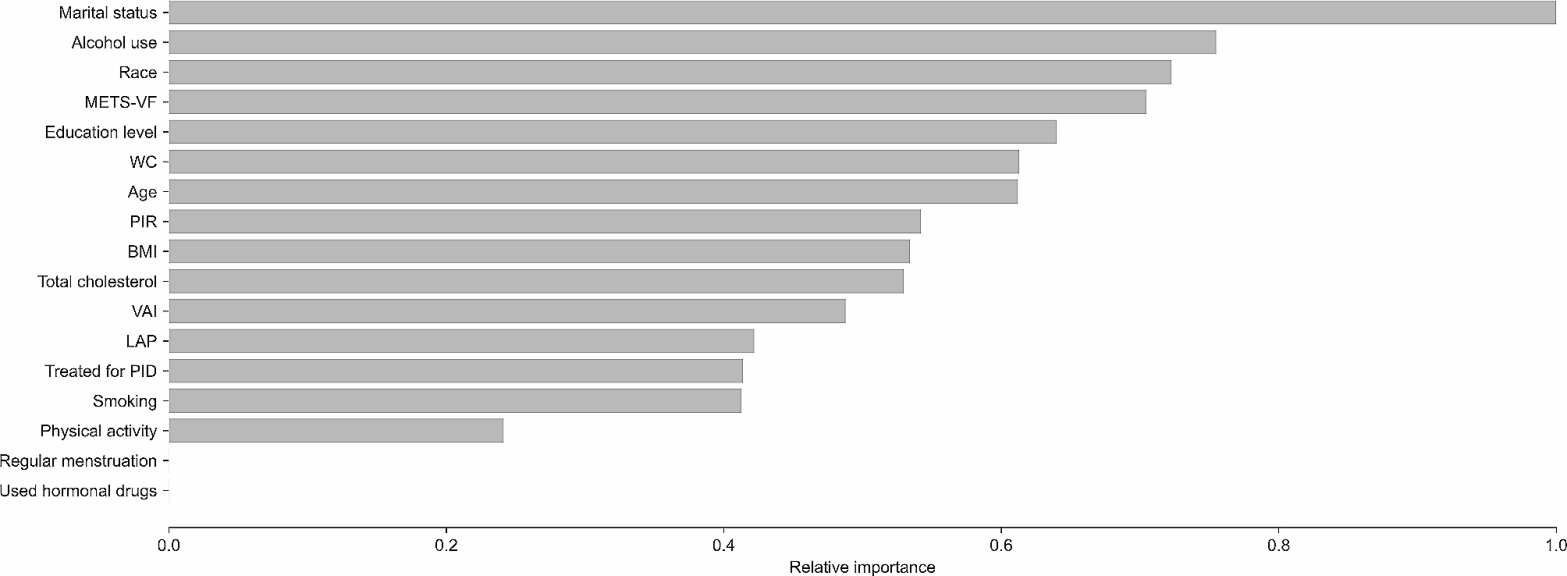
Results of the XGBoost algorithm. XGBoost, eXtremeGradient Boosting

In order to determine the relative significance of particular variables for infertility, researchers modeled the XGBoost algorithm using machine learning in the present study (Fig. 3). The current findings of machine learning indicated that marital status, alcohol use, education level, METS-VF, and age were the five most important factors. Furthermore, METS-VF was the most significant indicator of visceral lipid accumulation. The relative significance of METS-VF and WC is greater in comparison to the conventional BMI indicator. While the significance of LAP and VAI is lower compared to BMI.

### Correlation of different indicators of visceral lipid accumulation with central obesity and infertility

**Fig. 4:**
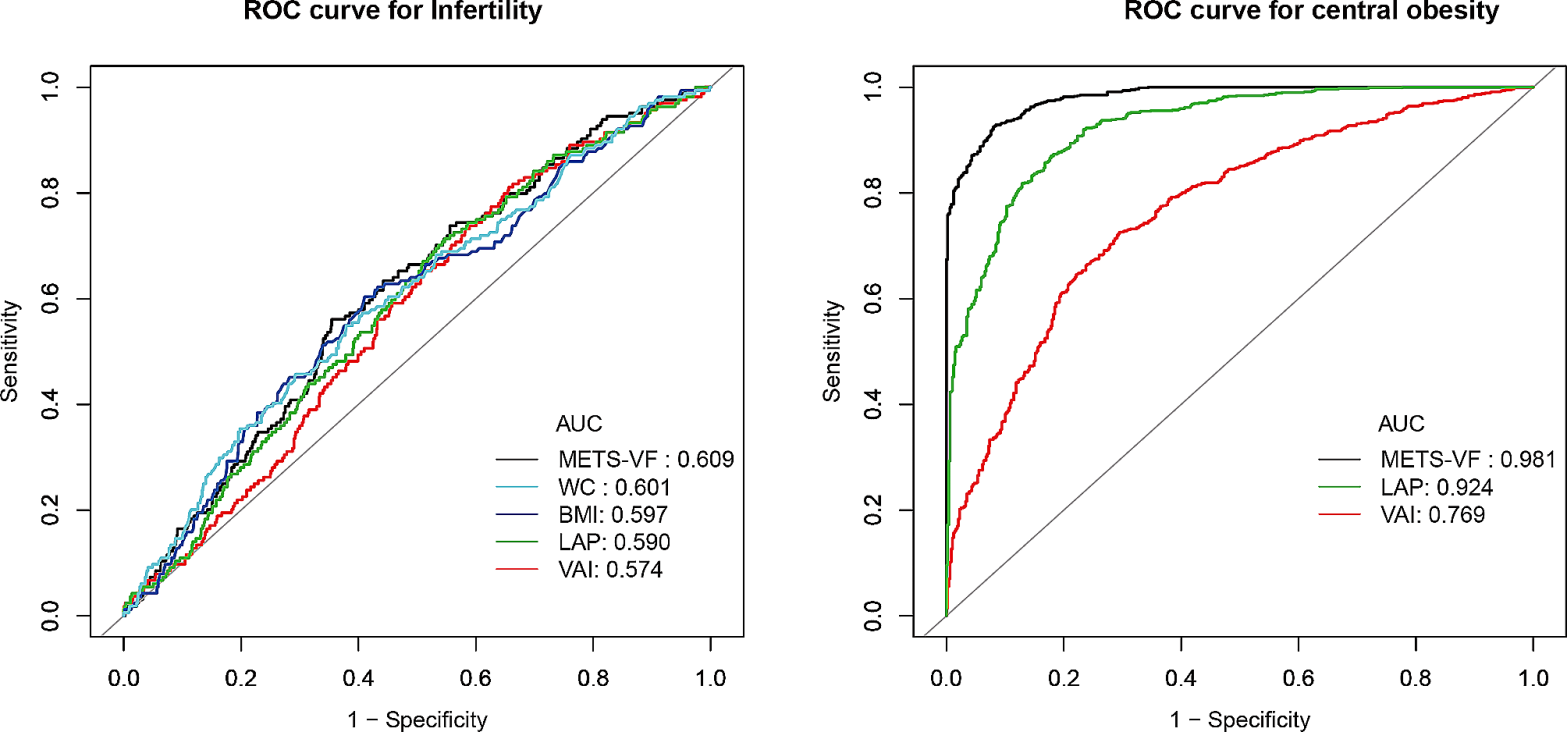
(**A**) ROC curves for different indicators to predict infertility. (**B**) ROC curves for different indicators to predict central obesity. ROC, Receiver Operating Characteristic; AUC, area under the curve

Table 5; Fig. 4 display the outcomes of the ROC curve analysis. In terms of the association with infertility, the AUC values of METS-VF, WC, LAP, and VAI were 0.6090 (95% CI: 0.5651,0.6530), 0.6013 (95% CI: 0.5550,0.6476), 0.5904 (95% CI: 0.5462,0.6346), and 0.5744 (95% CI: 0.5310,0.6179), respectively. The AUC of METS-VF had the highest value, but there was no statistically significant difference in the AUC values of the four indicators of visceral lipid accumulation (*P* > 0.05). Furthermore, there was no statistically significant disparity between the AUC values of the four indicators of visceral lipid accumulation and the AUC values of BMI (*P* > 0.05). Visceral obesity in women was classified as having a WC equal to or greater than 88 cm [32]. Regarding visceral obesity, the AUC values of METS-VF, LAP, and VAI were 0.9808 (95% CI: 0.9755,0.9861), 0.9097 (95% CI: 0.9097,0.9384), and 0.7690 (95% CI: 0.7428,0.7951), respectively. These values indicate that METS-VF is associated with central obesity to a greater extent than VAI and LAP (*P* < 0.0001).

**Table 5:** Comparison of ROC curves for different evaluation indicators of central obesity and infertility

Objects/Surrogates	Cutoff (Sensitivity, Specificity)	AUC (95% CI)	*P*-value	*P*-value (Compared with BMI)
**Infertility**				
METS-VF	6.7187 (0.5610, 0.6456)	0.6090	> 0.05	> 0.05
WC	99.9000 (0.5488, 0.6209)	0.6013		> 0.05
LAP	25.1156 (0.7134, 0.4529)	0.5904		> 0.05
VAI	0.7939 (0.8110, 0.3439)	0.5744		> 0.05
BMI	30.3500 (0.6037, 0.5893)	0.5965		NA
**Central obesity**				
METS-VF	6.2859 (0.9250, 0.9201)	0.9808	< 0.0001	NA
LAP	23.0380 (0.8696, 0.8217)	0.9097		
VAI	0.9894 (0.7257, 0.7049)	0.7690		

BMI, body mass index; WC, waist circumference; METS-VF, metabolism score for visceral fat; LAP, lipid accumulation product; VAI, visceral adiposity index; AUC, area under the curve; CI, confidence interval

### Sensitivity analysis

The original data had 554 participants excluded from the study due to missing corresponding covariates, which may reduced statistical test efficacy and bias associated with missing values. Therefore, the researchers employed multiple interpolations (MI), which were based on the R MI program, to estimate the impact of missing values. The R MI program was performed based on 5 repetitions and chained equations. The researchers performed multiple logistic regression analysis using the five sets of data obtained from MI. Next, the ORs, 95% CIs, and *P*-values of the multivariate logistic regression analyses were combined according to Rubin’s rule. To avoid the issue of excessively tiny impact value, the researchers opted to convert the units of WC utilized in the WC and LAP measurements to DM. As observed in Supplementary 2, the effect sizes of the interpolated data do not differ significantly from the original data, suggesting that missing data do not significantly affect the results.

## Discussion

This study examined the correlation between four indicators of visceral lipid accumulation and infertility in a sample of 1,301 participants. The results of the current investigation demonstrate a strong and positive correlation between all four indicators of lipid accumulation with infertility. Furthermore, these positive connections were consistent throughout different population settings. The four indicators showed comparable association with infertility, whereas METS-VF is associated with central obesity to a greater extent than VAI and LAP.

This study is the first to examine the correlations between indicators of visceral lipid accumulation and infertility using the NHANES database. Although there is an increasing number of researchers investigating the potential connection between visceral obesity and infertility, the research in this field is still restricted, and the existing findings are subject to controversy. There is compelling data indicating that an increase in WC is linked to the occurrence of negative outcomes during pregnancy, such as gestational diabetes, preeclampsia, and macrosomia [33]. A study conducted by Gao et al. examined 976 Chinese mother-child couples in a birth cohort study. The study revealed that having central obesity before conception can result in various negative pregnancy outcomes associated with obesity [34]. An independent study including 261 Turkish women revealed that having an abnormally wide WC both prior to and throughout pregnancy is a significant warning sign for the development of gestational diabetes [35]. A study based on 1,679 black women found that patients with higher WC and waist-to-hip ratio (WHR) had lower fertility [36]. Furthermore, the relationship of visceral adiposity with polycystic ovarian syndrome (PCOS), a prevalent factor contributing to female infertility, caught the attention of researchers. A case-control study conducted in Indonesia demonstrated that increased WC, hip circumference, and WHR considerably elevate the likelihood of developing PCOS in women [37]. Interestingly, Loy et al. obtained contrasting outcomes from a multiracial prospective cohort. No significant relationship between fertility and alternative indicators of central adiposity, including WHR, WHtR, and a body shape index (ABSI), was found [38]. The current investigation’s findings add new evidence to the correlation between indicators of central lipid accumulation and infertility. Specifically, after adjusting for all covariates, the prevalence of infertility in the highest tertiles of WC, METS-VF, LAP, and VAI was 202%, 248%, 234%, and 210% of that in the lowest tertiles, relatively. And the findings of the current investigation support the idea that central obesity is a dangerous element for infertility in women of reproductive age, although the underlying mechanisms remain unclear.

The global prevalence of obesity has reached alarming levels, affecting about 20% of women in America within the reproductive age group [39]. Multiple research investigations have demonstrated a correlation between obesity and disruptions in the hypothalamic-pituitary-gonadal (HPG) axis, resulting in impaired ovulation and anomalies in the menstrual cycle among females [23, 40]. This can be related to the correlation between visceral obesity and the development of leptin resistance. By influencing the activity of kisspeptin neurons, leptin governs the organism’s ability to reproduce by indirectly affecting the activity of the gonadotropin-releasing hormone (GnRH) [41]. Obesity clearly interferes with the ability of leptin to fulfill its usual physiological role. Additionally, women who are obese exhibit elevated levels of circulating free fatty acids. These fatty acids have the potential to accumulate outside of adipocytes, increasing reactive oxygen species (ROS). The presence of ROS can induce stress in both mitochondria and endoplasmic reticulum, ultimately leading to apoptosis. This lipotoxic mechanism is responsible for the destruction of non-adipocytes and may have detrimental effects on the processes of meiosis and cytoplasmic maturation of oocytes. These effects can subsequently hinder the fertilization process and compromise the developmental competence of pre-implantation embryos [42]. Furthermore, an abundance of free fatty acids can cause low-grade inflammation to persist in a variety of bodily tissues, including reproductive tissues. The abundance of mRNAs specific to oocytes is regulated by obesity-dependent changes in proinflammatory signaling, which leads to the disruption of normal embryonic development [42, 43]. Complex mechanisms mediate the relationship between visceral obesity and infertility, although such mechanisms remain incompletely elucidated, thus necessitating further research on the role of visceral obesity in women’s reproductive health to provide guidance for the prevention of female reproductive disorders.

It is worth mentioning that many previous research studies investigating the connection of overweight with infertility have mostly employed BMI as a tool to evaluate overweight. However, obesity can be categorized into two main types: generalized obesity and central obesity [44]. Furthermore, it is important to recognize that whereas BMI primarily assesses the overall degree of obesity in individuals [45], it might not sufficiently evaluate differences in body fat and muscle composition [46]. The research carried out by Li et al. provided confirmation that WC had a negative influence on the likelihood of a successful live delivery using assisted reproductive technologies, regardless of BMI [47]. Tang et al. examined 3,542 women in a metabolically healthy population and found no correlation between BMI and infertility [48]. Additionally, previous investigations have demonstrated that an elevated BMI is correlated with a better prognosis in advanced heart failure, and it is uncertain whether there is an obesity paradox in infertility, given the correlation between infertility in women and a higher likelihood of getting severe cardiovascular disease. Moreover, a few scholars have suggested that the existence of the obesity paradox can be attributed to the limitations of BMI [49–51]. Therefore, it is theoretically reasonable to assert that employing indications of visceral obesity is a more effective, precise, and cautious approach to evaluating female reproductive well-being. Magnetic resonance imaging (MRI) and computed tomography (CT) are universally acknowledged as the most reliable methods for evaluating visceral fat. However, their utilization as preferred metrics for assessing visceral fat is limited due to their high cost and the specialized nature of the procedures involved [52]. Therefore, employing novel indicators of lipid accumulation to evaluate infertility would be a more ideal approach. The indicators that are included are METS-VF, LAP, and VAI. It was discovered that there was a robust positive correlation between all four metrics and infertility. The ROC analysis confirmed that all four metrics could be utilized to identify infertility. However, the XGBoost modeling revealed that, in comparison to the conventional obesity metric BMI, only the METS-VF and WC exhibited a more pronounced ability to identify infertility. This can be related to the fact that VAI and LAP were not initially intended to evaluate visceral fat [53], which may explain why VAI and LAP are not as effective as BMI in identifying certain disorders associated with obesity. Moreover, the current investigation discovered that METS-VF exhibited a greater significance in relation to female infertility and proved to be a more effective measure for evaluating visceral obesity. This superiority extends to risk factors for infertility. A study conducted in China showed that METS-VF can be a dependable identifying indicator of type 2 diabetes in the Chinese population, due to other indicators of obesity, and is not affected by gender, age, or BMI [54]. An independent cohort study carried out in China revealed a direct association of METS-VF with the prevalence of hypertension. Furthermore, out of the six indicators analyzed, METS-VF demonstrated the highest AUC value, indicating that it could be a reliable and effective predictor of the risk of hypertension [55]. Furthermore, METS-VF was found to be strongly associated with the population’s risk of hyperuricemia by Liu XZ et al. [56]. The reason why the METS-VF has a superior predictive capacity is not well understood. Researchers propose that the METS-VF includes the age of the individual, which is a significant risk factor for numerous diseases that are not considered by other evaluation indicators. Furthermore, METS-VF includes lipid biomarkers that not only indicate the extent of fat buildup but also provide insights into the functionality of lipid cells. Moreover, insulin resistance, a constituent of the METS-VF, is commonly seen in conjunction with obesity and abnormal tolerance for glucose, both of which are risk factors together known as metabolic syndrome [57, 58]. Metabolic syndrome is not only linked to female fertility but also to the onset of various other diseases [59]. While the other indicators, such as BMI, WC, and VAI only provide a rough estimate of visceral adipose tissue content based on the distribution of body fat and do not precise depictions of the impact of fat on metabolism, the METS-VF effectively captures the influence of visceral adipose tissue on metabolism [60]. In summary, the current work has demonstrated that METS-VF is a reliable indicator of both female infertility and visceral obesity. Therefore, it is imperative to determine the METS-VF values of women experiencing infertility in order to enhance the efficacy of their treatment and the overall management of their condition.

## Study strengths and limitations

When compared to earlier research, this one has a number of remarkable strengths. Initially, researchers used a nationally representative sample of NHANES participants as the study population, adjusting the confounding factors and demonstrating for the first time the correlation between novel indicators of visceral lipid accumulation and infertility. Second, it was novel to employ machine learning with XGBoost algorithmic modeling to ascertain the relative significance of particular variables. The current research presents fresh proof of the correlation between visceral fat and infertility as well as novel suggestions for the management and treatment of infertility going forward. Although this study has yielded significant findings, it is imperative to recognize the inherent constraints that are linked to it. The current investigation was limited by its cross-sectional methodology, which prevented the development of a causal association between indicators of visceral lipid accumulation and infertility. Consequently, it is imperative to conduct a more extensive prospective investigation in order to corroborate the findings and confirm the results of the current investigation. Furthermore, an examination of infertility was conducted utilizing a questionnaire, potentially introducing inaccuracies in the findings if participants had imperfect recollection of the material. Moreover, because of the limited data on women’s reproductive health provided by NHANES, we were unable to distinguish whether infertility was primary or secondary. Nor can we rule out the role of male factors in female infertility. What’s more, it cannot be ignored that this investigation was conducted exclusively on the U.S. population, whether its conclusion can be applied to populations in other regions remains to be further investigated.

## Conclusion

Elevated levels of WC, METS-VF, LAP, and VAI are connected with a greater possibility of female infertility. Given the ease and cost-efficiency of calculating these four indicators, it is advisable for physicians in clinical practice to conduct lipid testing and measure relevant physical indicators for women who may be experiencing infertility or have risk factors for infertility. This will allow for the calculation of the four indicators of visceral lipid accumulation and provide a comprehensive evaluation of the patient’s reproductive health. These findings present a valuable tool for early identification of infertility, particularly in the field of care of gynecology and obstetrics. Healthcare professionals can develop tailored dietary plans, exercise routines, and personalized treatment strategies for women experiencing infertility based on assessments of these four indicators of visceral lipid accumulation. The objective is to provide timely interventions for infertility, thereby improving the efficacy of infertility treatment and care. However, it’s worth noting that this study was conducted on a population of women in the United States. Therefore, further research is necessary to ascertain the generalizability of the findings to female populations in other regions.

### Electronic supplementary material

Below is the link to the electronic supplementary material.

Supplementary Material 1

Supplementary Material 2

## Data Availability

The original data were retrieved from https://wwwn.cdc.gov/nchs/nhanes/Default.aspx. The data used in the article has been submitted as an additional file.
