# Supplementary material for: Association between indicators of visceral lipid accumulation and infertility: a cross-sectional study based on U.S. women
Source: Lipids Health Dis. 2024 Jun 13;23:186. doi: 10.1186/s12944-024-02178-x (PMC11170861; doi:10.1186/s12944-024-02178-x)
Supplement: Supplementary file 1 — Supplementary Material 1 [file 12944_2024_2178_MOESM1_ESM.docx]

**Supplementary 1.** Relationship of WC, LAP with Infertility after conversion units.

| **Exposure** | **OR (95% CI), *P*-value** | | |
| --- | --- | --- | --- |
|  | **Model 1** | **Model 2** | **Model 3** |
| WC | 1.18 (1.09, 1.28), <0.0001 | 1.16 (1.07, 1.26), 0.0004 | 1.18 (1.09, 1.29), 0.0001 |
| LAP | 1.05 (1.02, 1.08), 0.0010 | 1.04 (1.01, 1.08), 0.0070 | 1.06 (1.03, 1.10), 0.0005 |

Model 1, crude model;

Model 2, adjusted for age, race;

Model 3, adjusted for age, race, education, marital status, family income to poverty ratio, total cholesterol, smoking, alcohol use, physical activity, menstrual status, treatment of pelvic inflammatory disease, and use of hormonal medications.

OR, Odds ratio; CI, Confidence Interval; WC, waist circumference; LAP, lipid accumulation product.
